# Meta‐analysis on axillary lymph node metastasis rate in ductal carcinoma in situ with microinvasion

**DOI:** 10.1002/cam4.7413

**Published:** 2024-06-26

**Authors:** Xiongxiong Li, Can Zhou, Ting Xu, Yu Ren, Meng Li, Jin Shang

**Affiliations:** ^1^ Department of Breast Surgery Xi'an People's Hospital (Xi'an No. 4 Hospital) Xi'an China; ^2^ Department of Breast Surgery The First Affiliated Hospital of Xi'an Jiaotong University Xi'an China

**Keywords:** breast cancer, ductal carcinoma in situ with microinvasion, meta‐analysis, rate of axillary lymph nodes

## Abstract

**Objective:**

To address the question of axillary lymph node staging in ductal carcinoma in situ with microinvasion (DCIS‐MI), we retrospectively evaluated axillary lymph nodes metastasis (ALNM) rate in a cohort of postsurgical DCIS‐MI patients. By analyzing these data, we aimed to generate clinically relevant insights to inform treatment decision‐making for this patient population.

**Methods:**

A systematic search was conducted on PubMed, Web of Science, Embase, The Cochrane Library, CNKI, Wanfang Database, Wipe, and China Biomedical Literature Database to identify relevant publications in any language. All the analyses were performed using Stata 16.0 software.

**Results:**

Among the 28 studies involving 8279 patients, the pooled analysis revealed an ALNM rate of 8% (95% CI, 7% to 10%) in patients with DCIS‐MI. Furthermore, the rates of axillary lymph node macrometastasis, micrometastasis, and ITC in patients with DCIS‐MI were 2% (95% CI, 2% to 3%), 3% (95% CI, 2% to 4%), and 2% (95% CI, 1% to 3%), respectively. Moreover, 13 studies investigated the non‐sentinel lymph node (Non‐SLN) metastasis rate, encompassing a total of 1236 DCIS‐MI cases. The pooled analysis identified a Non‐SLN metastasis rate of 33% (95% CI, 14% to 55%) in patients with DCIS‐MI.

**Conclusion:**

The SLNB for patients with DCIS‐MI is justifiable and could provide a novel therapeutic basis for systemic treatment decisions.

## 1 INTRODUCTION

With the widespread adoption in breast cancer screening, the incidence of ductal carcinoma in situ (DCIS) was accounting for 25% of all new cancer cases,^1^ with 10% of them presenting in combination with ductal carcinoma in situ with microinvasion (DCIS‐MI).^2^ According to the 8th edition of the American Joint Committee on Cancer (AJCC) staging system for breast cancer, DCIS‐MI is defined by the presence of cancer cells that have penetrated the basement membrane and invaded adjacent tissues, with the invasive foci or any combination of foci limited to a maximum diameter of 1 mm (≤1 mm).^3^ Kotani et al.^4^ reported that 20% of initial DCIS diagnoses made via stereotactic vacuum‐assisted biopsy were subsequently upstaged to invasive ductal carcinoma (IDC) or DCIS‐MI. Similarly, findings from Flanagan^5^ revealed that 37% of patients initially diagnosed with DCIS‐MI through core biopsy were later upstaged to IDC. Consequently, the definitive diagnosis of DCIS or DCIS‐MI relied on postoperative pathological examination, prompting the recommendation of sentinel lymph node biopsy (SLNB) for patients diagnosed with DCIS or DCIS‐MI through preoperative puncture. However, there is no consensus regarding the necessity of axillary lymph node staging for patients diagnosed DCIS‐MI postoperatively.

Theoretically, DCIS‐MI posed a risk of axillary lymph node metastasis (ALNM). Although small sample sizes and retrospective clinical studies suggested a variable rate of 0%–25% of ALNM^6^, ^7^ for patients with DCIS‐MI, there are significant discrepancies in the reported figures. This divergence has led to considerable debate concerning the rate of ALNM and the appropriate management of axillary lymph nodes in DCIS‐MI cases. According to the NCCN^8^ guidelines, axillary lymph node status is a crucial factor influencing decisions regarding a patient's adjuvant treatment.

We conducted a meta‐analysis to investigate the ALNM rate in patients with DCIS‐MI following surgical resection. By analyzing relevant literature on DCIS‐MI and ALNM, we aimed to provide valuable insights to inform treatment decision‐making for this patient population.

## 2 METHODS

### 2.1 Inclusion and exclusion criteria

The inclusion criteria for the eligible studies were as follows, having: (1) the definition for DCIS‐MI matched AJCC criteria (invasion lesions ≤1 mm); (2) axillary lymph node staging (SLNB and/or axillary lymph nodes dissection) for patients with DCIS‐MI; (3) nodal metastases categorizing as macrometastasis (pN1, metastases>2 mm in size), micrometastasis (pN1mi, metastases 0.2 to 2 mm in size and/or >200 cells), or isolated tumor cells (ITC pN0i+, <0.2 mm in size, and/or ≤200 cells).

The exclusion criteria were as follows, having: (1) secondary analysis literature; (2) DCIS‐MI after neoadjuvant therapy; (3) less than 10 patients sample size; (4) inability to obtain full text or duplicate literature; (5) non‐Chinese or non‐English literature.

### 2.2 Search strategy

From the beginning until December 2023, a comprehensive search was conducted in various electronic databases, including PubMed, the Cochrane Library, Embase, Web of Science, China National Knowledge Infrastructure (CNKI), Chinese Scientific Journals Full‐Text Database (VIP), Chinese Biological Medicine Digest (CBMD), and Wanfang Database by two independent reviewers (Xiongxiong Li and Ting Xu). Additionally, ClinicalTrials.gov and Google Scholar were explored to uncover any unpublished or potential studies. The search criteria comprised a combination of Free Words and Medical Subject Headings (MeSH) terms, specifically focusing on “Sentinel node biopsy”, “SlNB”, “sentinel lymph node biopsy”; “Breast Neoplasm”, “Breast Tumor”, “Breast Cancer”, “Breast Malignant Neoplasm”, “Breast Carcinoma”; “Microinvasive”. All potentially eligible studies were considered for review, regardless of language or date of publication.

### 2.3 Data extraction

Using EndNote for literature management, after removing duplicates, relevant literature matching the inclusion and exclusion criteria was selected by reviewing the title, abstract, and full text. Excel software was then utilized to extract basic information from the literature, including the first author, research type, sample size, year of publication, country, the staining method of SLNB, lymph node histological examination method, data sources, study quality, region, tumor grading classification, classification of DCIS combined with necrosis, classification of DCIS types, and classification of vascular infiltration. Two reviewers (Xiongxiong Li and Ting Xu) independently extracted the relevant information, with any discrepancies resolved by involving a third examiner to reach a consensus.

### 2.4 Assessment of the risk of bias

Two reviewers (Xiongxiong Li and Ting Xu) independently assessed the risk of bias for each study using the criteria recommended by the Agency for Healthcare Research and Quality (AHRQ) for the cross‐sectional studies, with a third reviewer resolving any discrepancies.^9^ Scores of “yes,” “no,” and “unclear” were assigned 1, 0, and 0 points, respectively, with a maximum score of 11 points. Scores of 0–3 points were considered low quality, 4–7 points were considered medium quality, and 8–11 points were considered high quality.^10^ The risk of bias assessments is presented in Table 2.

### 2.5 Data synthesis and analysis

In assessing the rate of lymph node metastasis, including ITC metastasis, micrometastasis, macrometastasis, and non‐sentinel lymph node (Non‐SLN) metastasis in axillary lymph nodes of DCIS‐MI, statistical measures were employed, with a 95% confidence interval (95% CI) provided. The calculation of the ALNM rate began with the Freeman–Tukey Double arcsine transformation of the data. Subsequently, a chi‐squared inspection (with an inspection level of *α* = 0.05), combined with a *Q*‐test and *I* ^2^ quantitative assessment, was utilized to determine heterogeneity among research results. In cases where *I* ^2^ > 50% and *p* < 0 05, indicating significant heterogeneity among different studies, a random effects model was employed for merge analysis. Conversely, if *I* ^2^ < 50% and *p* > 0 05, indicating no significant heterogeneity between studies, a fixed effects model was performed for merge analysis. Sensitivity analysis was conducted to explore the impact of individual studies on the incidence of ALNM in DCIS‐MI patients by systematically removing them one by one. The calculation of ALNM rates allowed for comparisons across different subgroups, including study types, SLNB development staining techniques, lymph node histological examination, data sources, study quality, region‐wise, tumor grading classification, classification of DCIS combined with necrosis, classification of DCIS types, classification of vascular infiltration, and time span. Publication bias was assessed through funnel plots and Egger's tests. Statistical analyses were performed using the “meta” and “metaprop” programs in Stata 16.0 software.

## 3 RESULTS

### 3.1 Literature selection results

**FIGURE 1:**
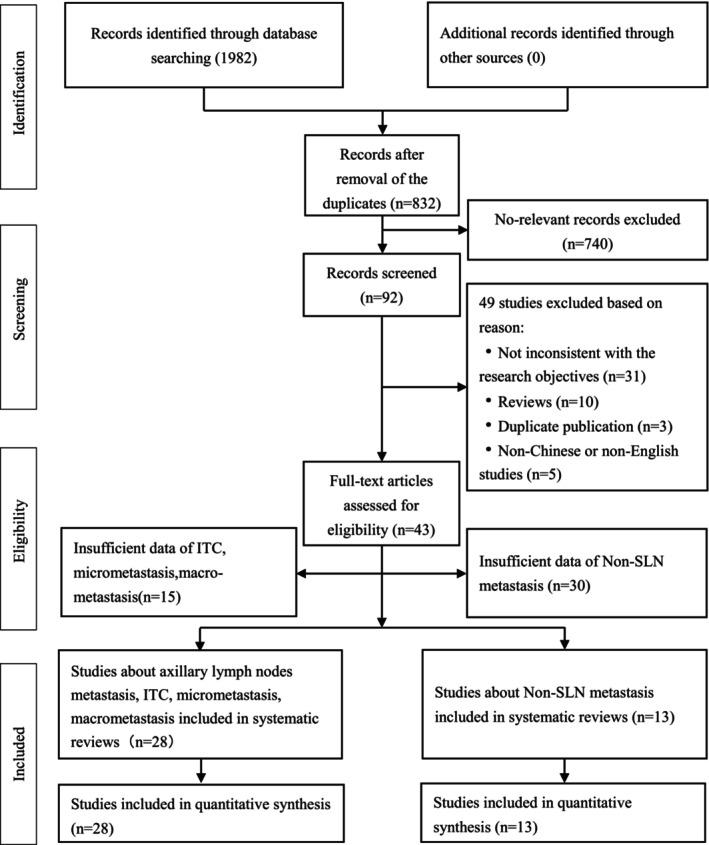
PRISMA‐guided flow diagram of study selection.

The initial literature search yielded 1982 records, with 1150 excluded due to duplicate items. After a thorough screening of the 832 articles, 740 articles were excluded due to the irrelevant content for the systematic review, leaving 92 studies for full‐text reading. Of these, 49 studies were excluded because they did not report any outcome measures. According to the inclusion and exclusion criteria, 28 studies were included to evaluate the rates of ALNM, ITC metastasis rate, micrometastasis, and macrometastasis in DCIS‐MI. Additionally, 13 studies were included to analyze the positivity rate of Non‐SLN metastasis in DCIS‐MI. A detailed flowchart of the literature retrieval process is illustrated in Figure 1.

### 3.2 Included studies and Characteristics

Table 1 provides a summary of the baseline characteristics of the included studies. In the analysis, 28 studies comprising 8279 patients with DCIS‐MI who underwent evaluation for ALNM, macrometastasis, micrometastasis, and ITC were identified. Geographically, the studies originated from Asia (*n* = 8), Europe (*n* = 8), and North America (*n* = 12). Regarding study design, 6 studies employed a prospective approach, while the remaining 22 studies were retrospective. Staining techniques for SLNB development varied across the studies, with 3 studies employing Tc‐99 m sulfur colloid, 11 ones using Tc‐99 m sulfur colloid and blue dye, and 14 ones lacking information on the staining technique. Histological examination methods for SLNB were diverse as well, with 10 studies employing H&E and IHC, 4 studies using only H&E, 7 ones using only IHC, and 7 studies lacking information on SLNB histological examination (Table 1).

**TABLE 1:** Characteristics of studies included in the systematic review.

Study	Country	Age (median)	Study design	Study time	SLNB		The number of nodes						DCIS‐MI
					Staining technique	Histological examination	ITC	MICRO	MACRO	Total	Non‐SLN	ALND	
Klauber 2000^11^	USA	51 (31–80)	Prospective	1997–1999	Both1	Both2	0	2	1	3	0	3	31
Zavagno 2006^12^	Italy	58 (26–77)	Retrospective	1991–2004	Tc‐99 m	Both2	0	1	3	4	4	4	43
Katz 2006^13^	USA	58 (33–86)	Retrospective	1998–2003	Both1	Both2	0	1	1	2	1	1	21
Guth 2008^14^	USA	Mean 57.6	Retrospective	1991–2006	Both1	IHC	2	0	3	5	1	2	44
Pimiento 2011^15^	USA	59 (30–89)	Retrospective	1996–2009	Both1	Both2	3	2	4	9	2	6	87
Ko 2012^16^	Korea	47 (24–82)	Retrospective	1989–2008	Both1	IHC	6	12	4	22	NR	NR	293
Meretoja 2012^17^	Finland	56 (39–84)	Prospective	2001–2010	Both1	Both2	1	2	4	7	1	6	34
Lyons 2012^18^	USA	NR	Retrospective	1996–2004	Both1	Both2	6	5	3	14	5	6	112
Sun 2012^19^	China	NR	Retrospective	2002–2011	Both1	H&E	4	6	1	11	5	11	88
Kapoor 2013^20^	USA	52 (30–92)	Prospective	1995–2010	NR	NR	4	4	1	9	NR	NR	45
Hanna 2014^21^	USA	56 (31–83)	Retrospective	1994–2012	NR	H&E	7	0	0	7	0	4	81
Ozkan 2014^22^	Turkey	51 (22–75)	Retrospective	2000–2008	Both1	Both2	0	1	1	2	NR	NR	34
Masten 2014^23^	USA	54 (27–84)	Prospective	1997–2010	NR	Both2	0	26	6	31	NR	NR	414
Orzalesi 2015^24^	Italy	NR	Prospective	1992–2014	Tc‐99 m	IHC	10	3	5	18	1	6	126
Wang 2016^25^	China	51 (30–81)	Retrospective	2010–2015	NR	H&E	0	2	8	10	NR	NR	182
Lillemoe 2017^26^	USA	57 (31–88)	Retrospective	2001–2015	NR	H&E	0	3	1	4	NR	NR	260
Kim 2018^27^	Korea	Mean 50	Retrospective	2003–2014	NR	IHC	0	2	2	4	NR	NR	110
Costarelli 2018^28^	Italy	Mean 56.4	Prospective	2011–2016	NR	IHC	0	5	20	25	NR	NR	203
Magnoni 2019^29^	Italy	NR	Retrospective	1998–2010	Tc‐99 m	Both2	12	14	5	31	16	31	257
Holm 2019^7^	Denmark	NR	Retrospective	2002–2015	Both1	NR	9	18	23	50	2	20	233
Bertozzi 2019^30^	Italy	Mean 58.8	Retrospective	2002–2016	NR	NR	5	3	3	11	NR	NR	84
Hotton 2019^6^	France	NR	Retrospective	2003–2017	NR	Both2	0	0	0	0	NR	NR	27
Phantana 2019^31^	USA	57 (40–85)	Retrospective	2006–2017	NR	NR	0	1	0	1	NR	NR	47
Zhang 2019^32^	China	48 (23–77)	Retrospective	2004–2016	Both1	NR	0	1	2	3	0	3	79
Fan 2020^33^	USA	NR	Retrospective	2012–2015	NR	NR	68	37	39	114	NR	NR	2609
Zhang 2021^34^	China	Mean 46	Retrospective	2012–2019	NR	IHC	2	5	2	9	NR	NR	72
Paik 2022^35^	Korea	NR	Retrospective	1996–2020	NR	NR	23	29	69	121	NR	NR	2620
Hacking 2022^36^	USA	Mean 56.7	Retrospective	2010–2020	NR	IHC	1	0	1	2	NR	NR	43

*Note*: Both1: Tc‐99 m (technetium‐99 m sulfur colloid) + blue dye; Both2: immunohistochemical (IHC) + hematoxylin and eosin (HE); total: ITC (isolated tumor cells micrometastasis) + micro (micrometastasis) + macro (macrometastasis).

Abbreviations: ALND, axillary lymph node dissection; DCIS‐MI, ductal carcinoma in situ with microinvasion; Non‐SLN, non sentinel lymph nodes; NR, not reported; SLNB, sentinel lymph node biopsy.

Among the 13 studies investigating Non‐SLN metastasis and encompassing 1236 DCIS‐MI cases, the majority originated from North America (*n* = 6), followed by Europe (*n* = 5) and Asia (*n* = 2). Regarding study design, 3 studies were prospective, while 10 adopted a retrospective approach. Staining techniques for SLNB development varied, with 3 studies using Tc‐99 m sulfur colloid, 9 employing Tc‐99 m sulfur colloid and Blue dye, and 1 study lacking information on the staining technique. Histological examination methods for SLNB included 7 studies with H&E and IHC, 2 ones with only H&E, 2 ones with only IHC, and 2 studies lacking information on SLNB histological examination (Table 1).

### 3.3 Quality assessment of included studies

The quality grades for each study are presented in Table 2, and notably, no articles received a low‐quality rating. Thirteen studies met high‐quality criteria for evaluating ALNM, macrometastasis, micrometastasis, and ITC rates in DCIS‐MI. An additional 15 studies were classified as moderate quality (Table 2). Similarly, following a comprehensive quality assessment, 7 studies were deemed high quality for analyzing the Non‐SLN metastasis rate in DCIS‐MI, with 6 studies rated as moderate quality (Table 2).

**TABLE 2:** Quality evaluation of studies included in the systematic review.

Study	①	②	③	④	⑤	⑥	⑦	⑧	⑨	⑩	⑪	Quality score	Quality grade
Klauber 2000^11^	Y	Y	Y	Y	U	Y	Y	N	U	Y	N	7	M
Zavagno 2006^12^	Y	Y	Y	Y	U	Y	Y	N	U	Y	N	7	M
Katz 2006^13^	Y	Y	Y	Y	U	Y	Y	N	U	Y	Y	8	H
Guth 2008^14^	Y	Y	Y	Y	U	Y	Y	N	U	Y	N	7	M
Pimiento 2011^15^	Y	Y	Y	Y	U	Y	Y	N	U	Y	Y	8	H
Ko 2012^16^	Y	Y	Y	Y	U	Y	Y	N	U	Y	N	7	M
Meretoja 2012^17^	Y	Y	Y	Y	U	Y	Y	N	U	Y	Y	8	H
Lyons 2012^18^	Y	Y	Y	Y	U	Y	Y	N	U	Y	Y	8	H
Sun 2012^19^	Y	Y	Y	Y	U	Y	Y	N	U	Y	N	7	M
Kapoor 2013^20^	Y	Y	Y	Y	U	U	Y	N	U	Y	N	6	M
Hanna 2014^21^	Y	Y	Y	Y	U	Y	Y	N	U	Y	Y	8	H
Ozkan 2014^22^	Y	Y	Y	Y	U	Y	Y	N	U	Y	Y	8	H
Masten 2014^23^	Y	Y	Y	Y	U	Y	Y	N	U	Y	Y	8	H
Orzalesi 2015^24^	Y	Y	Y	Y	U	Y	U	N	U	Y	Y	8	H
Wang 2016^25^	Y	Y	Y	Y	U	Y	U	N	U	Y	N	7	M
Lillemoe 2017^26^	Y	Y	Y	Y	U	Y	Y	N	U	Y	Y	8	H
Kim 2018^27^	Y	Y	Y	Y	U	Y	Y	N	U	Y	Y	8	H
Costarelli 2018^28^	Y	Y	Y	Y	U	Y	Y	N	U	Y	N	7	M
Magnoni 2019^29^	Y	Y	Y	Y	U	Y	Y	N	U	Y	Y	8	H
Holm 2019^7^	Y	Y	Y	Y	U	U	Y	N	U	Y	N	6	M
Bertozzi 2019^30^	Y	Y	Y	Y	U	U	Y	N	U	Y	Y	7	M
Hotton 2019^6^	Y	Y	Y	Y	U	Y	U	N	U	Y	Y	8	H
Phantana 2019^31^	Y	Y	Y	Y	U	U	Y	N	U	Y	Y	7	M
Zhang 2019^32^	Y	Y	Y	Y	U	U	Y	N	U	Y	N	6	M
Fan 2020^33^	Y	Y	Y	Y	U	U	Y	N	U	Y	N	6	M
Zhang 2021^34^	Y	Y	Y	Y	U	Y	Y	N	U	Y	N	7	M
Paik 2022^35^	Y	Y	Y	Y	U	U	Y	N	U	Y	N	6	M
Hacking 2022^36^	Y	Y	Y	Y	U	Y	Y	N	U	Y	Y	8	H

*Note*: ① Define the source of information (survey, record review); ② list inclusion and exclusion criteria for exposed and unexposed subjects (cases and controls) or refer to previous publications; ③ indicate the time used for identifying patients; ④ indicate whether subjects were consecutive if not population‐based; ⑤ indicate if evaluators of subjective components of study were masked to other aspects of the participants; ⑥ describe any assessments undertaken for quality assurance purposes (e.g., test/retest of primary outcome measurements); ⑦ explain any patient exclusions from analysis; ⑧ describe how confounding was assessed and/or controlled; ⑨ if applicable, explain how missing data were handled in the analysis; ➉ summarize patient response rates and completeness of data collection; ⑪ clarify what follow‐up, if any, was expected and the percentage of patients for which incomplete data or follow‐up was obtained.

Abbreviations: H, high quality; M, medium quality; N, no; U, unclear; Y, yes.

### 3.4 The ALNM rate for patients with DCIS‐MI

**FIGURE 2:**
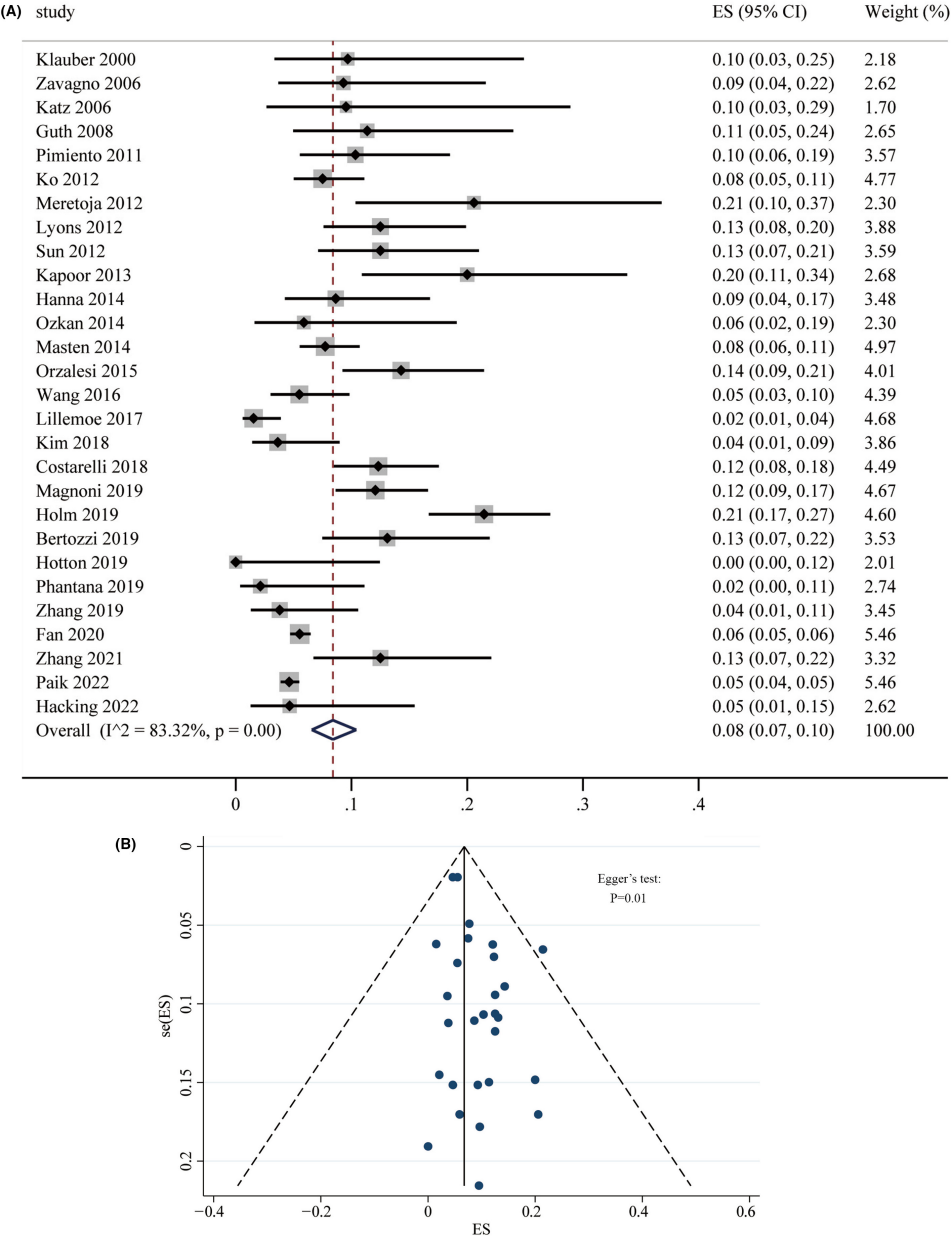
(A) Forest plot and (B) funnel plot of axillary lymph node metastasis rate in DCIS‐MI.

The pooled ALNM rate in patients with DCIS‐MI was high, reaching 8% (95% CI: 7% to 10%). Due to significant heterogeneity among studies (*I* ^2^ = 83.32%, *p* = 0.00) (Figure 2A), a random effects model was employed for the meta‐analysis. However, potential publication bias was identified. Although the funnel plots (Figure 2B) appeared symmetrical and fell within the 95% CI, Egger's test indicated a statistically significant bias (*p* = 0.01). Subgroup analysis revealed significant differences (*Q* = 14.28, *p* < 0.01) in ALNM rate based on the staining technique used for SLNB and by geographic region (*Q* = 9.91, *p* = 0.01) (Table 3).

**TABLE 3:** Axillary lymph node positivity rates in different subgroups of DCIS‐MI.

	Axillary lymph nodes of DCIS‐MI																
	No. studies	Metastasis rate (95% CI)	*Q*‐value	*p*	Macrometastasis rate (95% CI)	*Q*‐value	*p*	Micrometastases rate (95% CI)	*Q*‐value	*p*	ITC metastasis rate (95% CI)	*Q*‐value	*p*	No. studies	Non‐SLN metastasis rate (95% CI)	*Q*‐value	*p*
Study type			4.94	0.03		0.91	0.34		3.51	0.06		0.21	0.65			3.04	0.08
Retrospective	22	0.07 (0.06–0.09)			0.02 (0.01–0.03)			0.02 (0.01–0.03)			0.01 (0.01–0.03)			10	0.41 (0.17–0.68)		
Prospective	6	0.12 (0.08–0.17)			0.04 (0.01–0.08)			0.04 (0.02–0.07)			0.02 (0.00–0.08)			3	0.11 (0.00–0.36)		
SLNB development staining technique			14.28	0.00					7.79	0.02		5.9	0.05			4.04	0.13
Tc‐99 m sulfur colloid	3	0.12 (0.09–0.16)			0.03 (0.01–0.06)	2.88	0.24	0.04 (0.02–0.06)			0.04 (0.01–0.09)			3	0.57 (0.16–0.94)		
Both 1	11	0.11 (0.07–0.15)			0.03 (0.02–0.06)			0.04 (0.02–0.06)			0.02 (0.01–0.04)			9	0.28 (0.07–0.54)		
Not mentioned	14	0.06 (0.04–0.08)			0.02 (0.01–0.03)			0.02 (0.01–0.03)			0.01 (0.0–0.02)			1	0.00 (0.00–0.49)		
SLNB histologicaL examination			1.5	0.68		3.14	0.37		10.33	0.02		0.05	1			8.8	0.03
Both2	10	0.09 (0.07–0.12)			0.02 (0.01–0.03)			0.04 (0.03–0.06)			0.01 (0.00–0.04)			7	0.52 (0.23–0.81)		
HE	4	0.06 (0.02–0.12)			0.01 (0.00–0.04)			0.02 (0.00–0.04)			0.02 (0.00–0.07)			2	0.29 (0.07–0.57)		
IHC	7	0.09 (0.06–0.13)			0.04 (0.01–0.07)			0.02 (0.01–0.04)			0.02 (0.00–0.05)			2	0.22 (0.00–0.63)		
Not mentioned	7	0.09 (0.05–0.13)			0.03 (0.01–0.05)			0.03 (0.01–0.04)			0.02 (0.01–0.04)			2	0.05 (0.00–0.22)		
Data sources			0.05	0.82		0.71	0.40		0.47	0.49		0.76	0.38			0.91	0.34
Multicenter	9	0.08 (0.05–0.12)			0.03 (0.02–0.05)			0.03 (0.01–0.04)			0.01 (0.00–0.03)			4	0.48 (0.15–0.81)		
Single‐center	19	0.08 (0.06–0.11)			0.02 (0.01–0.03)			0.03 (0.02–0.04)			0.02 (0.00–0.04)			9	0.24 (0.04–0.51)		
Studies quality			0.37	0.54		4.27	0.04		0.05	0.83		0.49	0.48			0.10	0.76
Medium quality	15	0.09 (0.07–0.12)			0.03 (0.02–0.05)			0.03 (0.02–0.04)			0.01 (0.00–0.03)			6	0.30 (0.02–0.68)		
High quality	13	0.08 (0.05–0.11)			0.01 (0.01–0.02)			0.02 (0.01–0.04)			0.02 (0.00–0.05)			7	0.37 (0.13–0.63)		
Region wise			9.91	0.01		7.10	0.03		2.17	0.34		4.95	0.08			0.05	0.98
Asia	8	0.06 (0.04–0.08)			0.02 (0.02–0.03)			0.03 (0.01–0.05)			0.01 (0.00–0.02)			2	0.32 (0.07–0.62)		
Europe	8	0.13 (0.09–0.17)			0.05 (0.02–0.08)			0.04 (0.02–0.06)			0.03 (0.01–0.06)			5	0.37 (0.09–0.69)		
America	12	0.07 (0.05–0.10)			0.01 (0.00–0.02)			0.02 (0.01–0.04)			0.02 (0.00–0.04)			6	0.35 (0.02–0.77)		
Tumor grading classification			8.67	0.12		7.26	0.20		5.86	0.32		12.82	0.03			1.27	0.94
I, II, III	5	0.09 (0.05–0.14)			0.02 (0.01–0.03)			0.05 (0.03–0.07)			0.01 (0.00–0.06)			4	0.41 (0.03–0.85)		
I, II, III, unknown	2	0.13 (0.08–0.19)			0.03 (0.01–0.07)			0.02 (0.00–0.06)			0.06 (0.02–0.10)			2	0.25 (0.00–0.74)		
I, II and III	13	0.07 (0.05–0.10)			0.03 (0.01–0.04)			0.02 (0.01–0.03)			0.01 (0.00–0.02)			2	0.33 (0.21–0.47)		
I, II and III and unknown	3	0.09 (0.06–0.12)			0.01 (0.00–0.03)			0.02 (0.00–0.06)			0.05 (0.01–0.10)			2	0.45 (0.13–0.79)		
III	2	0.09 (0.04–0.15)			0.04 (0.01–0.09)			0.02 (0.00–0.06)			0.02 (0.00–0.06)			1	0.33 (0.10–0.70)		
Not mentioned	3	0.14 (0.08–0.21)			0.04 (0.01–0.09)			0.04 (0.00–0.13)			0.04 (0.00–0.11)			2	0.12 (0.00–0.63)		
DCIS combined with necrosis classification			0.59	0.74		1.28	0.53		3.88	0.14		0.65	0.72			3.60	0.17
Yes and no	7	0.07 (0.03–0.11)			0.01 (0.00–0.04)			0.01 (0.00–0.03)			0.02 (0.00–0.07)			3	0.11 (0.00–0.42)		
Yes and no and unknown	1	0.10 (0.03–0.29)			0.05 (0.01–0.23)			0.05 (0.01–0.23)			0.00 (0.00–0.15)			1	1.00 (0.21–1.00)		
Not mentioned	20	0.09 (0.07–0.11)			0.03 (0.02–0.04)			0.03 (0.02–0.04)			0.02 (0.01–0.03)			9	0.37 (0.15–0.61)		
DCIS types classification			4.97	0.17		5.55	0.14		8.37	0.04		1.79	0.62			11.92	0.01
Comedo and non‐Comedo	6	0.04 (0.02–0.08)			0.01 (0.00–0.03)			0.01 (0.00–0.02)			0.01 (0.00–0.04)			2	0.08 (0.00–0.52)		
Comedo and non‐Comedo and unknown	1	0.10 (0.03–0.29)			0.05 (0.01–0.23)			0.05 (0.01–0.23)			0.00 (0.00–0.15)			1	1.00 (0.21–1.00)		
Four types	1	0.09 (0.04–0.22)			0.07 (0.02–0.19)			0.05 (0.01–0.12)			0.00 (0.00–0.08)			1	1.00 (0.51–1.00)		
Not mentioned	20	0.10 (0.07–0.12)			0.03 (0.02–0.04)			0.03 (0.02–0.05)			0.02 (0.01–0.03)			9	0.28 (0.11–0.48)		
Vascular infiltration classification			0.08	0.96		0.73	0.69		0.75	0.69		0.35	0.84			0.54	0.76
Yes and no	6	0.09 (0.04–0.17)			0.02 (0.00–0.06)			0.03 (0.01–0.07)			0.02 (0.00–0.05)			4	0.34 (0.00–0.90)		
Yes and no and unknown	4	0.08 (0.04–0.13)			0.02 (0.01–0.04)			0.02 (0.01–0.04)			0.03 (0.01–0.06)			2	0.25 (0.03–0.55)		
Not mentioned	18	0.08 (0.06–0.11)			0.03 (0.01–0.04)			0.02 (0.01–0.04)			0.01 (0.00–0.03)			7	0.38 (0.14–0.66)		
Time span			1.29	0.53		5.40	0.07		0.36	0.84		0.65	0.72			1.07	0.30
2000–2010	4	0.10 (0.05–0.16)			0.06 (0.02–0.10)			0.02 (0.00–0.06)			0.01 (0.00–0.03)				0.64 (0.01–1)		
2011–2020	21	0.09 (0.06–0.11)			0.02 (0.01–0.04)			0.03 (0.02–0.04)			0.02 (0.01–0.03)				0.28 (0.11–0.48)		
2021–2022	3	0.06 (0.02–0.12)			0.02 (0.02–0.03)			0.02 (0.00–0.06)			0.01 (0.00–0.03)				NR		

*Note*: *Q*‐value and *p* represented heterogeneity between sub‐groups; Both1: Tc‐99 m sulfur colloid + blue dye; Both2: immunohistochemical (IHC) + hematoxylin and eosin (HE).

Abbreviations: DCIS, ductal carcinoma in situ; DCIS‐MI, ductal carcinoma in situ with microinvasion; ITC, isolated tumor cells micrometastasis; Non‐SLN, non sentinel lymph nodes; NR, not reported.

### 3.5 The axillary lymph nodes macrometastasis, micrometastasis, and ITC metastasis rates for patients with DCIS‐MI

The pooled ALNM rates of macrometastasis, micrometastasis, and ITC was found to be 2% (95% CI, 2% to 3%), 3% (95% CI, 2% to 4%), and 2% (95% CI, 1% to 3%), respectively, with a random effects model performed for meta‐analysis in all cases owing to the heterogeneity of the *I* ^2^ values of 50% and more (Figures S1a, S2a, and S3a). No publication bias were found in the funnel plots for macrometastasis or ITC subgroups (Figures S1b and S3b), or the Egger's test results, rather than micrometastasis subgroup (Figure S2b).

In this study, macrometastasis showed significant subgroup differences in rate among regions (*Q* = 7.10, *p* = 0.03), while ITC (*Q* = 4.95, *p* = 0.08) and micrometastases (*Q* = 2.17, *p* = 0.34) did not demonstrate subgroup differences. Contrary, the SLNB staining development technique (*Q* = 7.79, *p* = 0.02) and SLNB histological examination (*Q* = 10.33, *p* = 0.02) showed significant group differences for micrometastases, while macrometastases and ITC did not (Table 3).

### 3.6 The Non‐SLN metastasis rate for patients with DCIS‐MI

**FIGURE 3:**
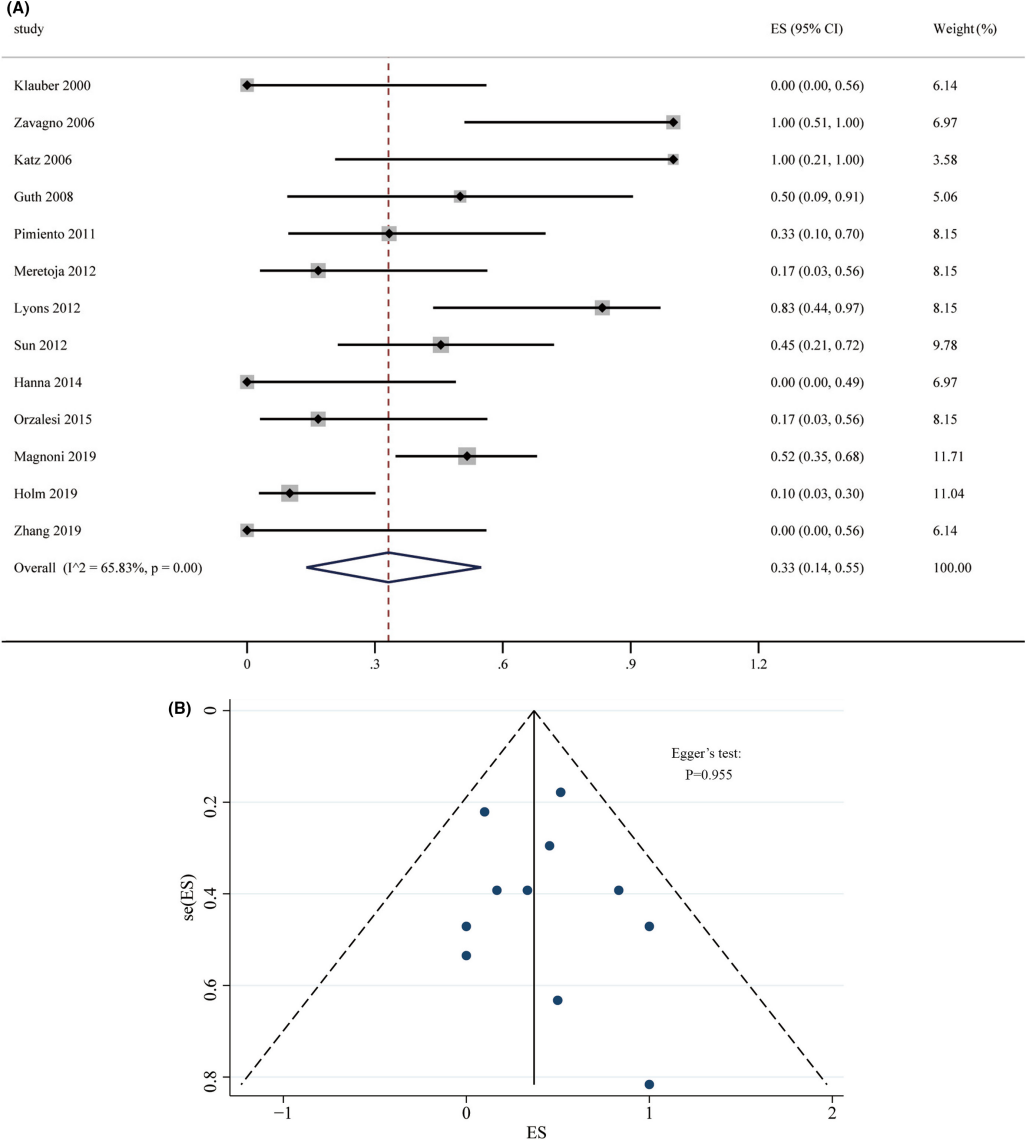
(A) Forest plot (B) funnel plot of Non‐SLN metastasis rate in DCIS‐MI.

The rate of Non‐SLN in patients with DCIS‐MI was high, at 33% (95% CI: 14% to 55%). Substantial heterogeneity was observed among studies (*I* ^2^ = 65.83%, *p* < 0.01) (Figure 3A). Funnel plot and Egger's test results did not suggest publication bias (Figure 3B). Interestingly, a higher rate of Non‐SLN metastasis was found in studies that utilized combined HE and IHC staining for histological examination compared to studies using HE or IHC alone (*Q* = 8.80, *p* = 0.03) (Table 3).

### 3.7 Sensitivity analysis

A sensitivity analysis was conducted by systematically removing individual studies one at a time. This analysis revealed that the pooled rates of ALNM, macrometastasis, micrometastasis, isolated tumor cells ITC, and Non‐SLN metastasis in DCIS‐MI patients ranged from 8% to 9%, 2% to 3%, 2% to 3%, 1% to 2%, and 27%–38%, respectively (Table S1). These rates remained consistent with the overall incidence rate, indicating the robustness of the study results.

## 4 DISCUSSION

### 4.1 Does DCIS‐MI require sentinel lymph node biopsy?

The incidence rate of patients with DCIS‐MI accounted for 0.68% to 2.4% of breast cancer cases.^37^ Given its low incidence rate, there existed a lack of consensus regarding the necessity of SLNB for DCIS‐MI (Table 4). Currently, there is no established guideline for managing axillary lymph node staging in patients with DCIS‐MI. Several studies have shown a lower systemic treatment rate for DCIS‐MI patients with SLN‐negative compared to ALNM‐positive patients **(**Table 5 **)**. This raises concerns about potential undertreatment in the SLN‐negative group, leading to a higher risk of recurrence and metastasis. Our study, encompassing 8279 DCIS‐MI patients, identified a pooled ALNM rate of 8%, consistent with the 7% rate reported in the preliminary study of 17,473 DCIS‐MI patients.^45^ More importantly, the axillary lymph node status was a crucial prognostic factor for breast cancer. Previous studies indicated that patients with ALNM have a worse prognosis compared to those without ALNM,^46^ including patients with axillary lymph node micrometastasis.^47^, ^48^, ^49^ Another study supported these findings, although patients with ITC metastasis in axillary lymph nodes had a similar prognosis to those with non‐metastasis, patients with micrometastases faced a 38% higher risk of recurrence than those with node‐negative patients.^50^

**TABLE 4:** Various literature opinions on whether sentinel lymph node biopsy was necessary in DCIS‐MI.

Study	Axillary lymph node metastasis rate, %	Opinions on whether sentinel lymph node biopsy was necessary
Klauber 2000^11^	9.68	Recommend
Zavagno 2006^12^	9.30	Recommend
Guth 2008^14^	11.36	Recommend
Fortunato 2008^38^	7.79	Individualized considering. Patients under 60 years old or histological grade III
Parikh 2010^39^	2.17	Not recommended
Pimiento 2011^15^	10.34	Recommend
Ko 2012^16^	7.51	Not recommended
Meretoja 2012^17^	20.59	Not recommended
Lyons 2012^18^	12.50	Recommend
Sun 2012^19^	12.50	Recommend
Kapoor 2013^20^	20.00	Only recommended when changing treatment strategies
Hanna 2014^21^	8.64	Not recommended
Masten 2014^23^	7.49	Only recommended when changing treatment strategies
Orzalesi 2015^24^	14.29	Individualized considering. Patients with DCIS with a diameter greater than 20 mm
Yi 2015^40^	3.30	Individualized considering. Patients undergoing mastectomy or DCIS with a diameter greater than 50 mm
Wang 2016^25^	5.49	Individualized considering. Patients over 50 years old or with a histological grade of III or a tumor diameter greater than 2.5 cm
Kim 2018^27^	3.64	Recommend
Magnoni 2019^29^	12.06	Not recommended
Holm 2019^7^	21.46	Individualized considering. Patients with age ≤49 or HER‐2 positive
Gooch 2019^41^	3.92	Individualized considering. Patients with lymphatic vessel invasion
Bertozzi 2019^30^	13.10	Recommend
Hotton 2019^6^	0.00	Not recommended
Phantana 2019^31^	2.13	Not recommended
Fan 2020^33^	4.37	Individualized considering. Patients with histological grade III
Strang 2020^42^	6.95	Recommend
Paik 2020^35^	4.62	Individualized considering. Patients under 50 years old or with HER‐2 positive or lymphatic vessel invasion or histological grade III
Hacking 2022^36^	4.65	Not recommended

**TABLE 5:** Various literature search results on the prognosis of DCIS‐MI.

Study	Macro	Follow‐up (months)	Outcomes	Systemic adjuvant therapy
Parikh 2010^38^	NR	107 (median)	10‐year DMFS 97.9%, 10‐year OS 95.7%	NR
Pimiento 2011^15^	4.60%	74.2 (median)	5‐year OS 94.2% (LN−), 5‐year OS 100% (LN+)	0% of negative SLN received systemic therapy and 100% of positive SLN received systemic adjuvant therapy
Meretoja 2012^17^	2.90%	50 (median)	1 patient occurred recurrence (LN−) and no patients occurred recurrence (LN+)	7.5% of negative SLN received systemic therapy and 100% of positive SLN received systemic adjuvant therapy
Lyons 2012^18^	2.70%	72 (median)	9 patients occurred recurrences (all in LN−, including 5 patients locoregional recurrences and 4 contralateral second primary cancers). No patients occurred recurrence (LN+)	38% of negative SLN received systemic therapy and 82% of positive SLN received systemic adjuvant therapy
Kapoor2013^20^	2.20%	83 (median)	3 patients occurred recurrences (LN−, 1 local, 1 distant, 1concurrent local and distant) and no patients occurred recurrence (LN+)	32% of negative SLN received systemic therapy and 89% of positive SLN received systemic adjuvant therapy
Hanna2014^21^	0.60%	37 (median)	3 patients occurred recurrences (LN−, 3 local) and no patients occurred recurrence (LN+)	NR
Masten2014^23^	1.40%	60 (median)	18 patients occurred recurrences (LN−, 14 local, 1 regional, 2 distant, and 1 concurrent local and distant) and no patients occurred recurrence (LN+). 5‐year OS 98.1% (LN−), 5‐year OS 100% (LN+)	The use of chemotherapy was more common among SLNB positive patients, 3%of SLNB‐negative patients received chemotherapy compared with 63% in the SLNB‐positive group (*p* < 0.01)
Lillemoe2017^26^	0.40%	55 (median)	No patients occurred recurrence	43.5% patients received endocrine treatment, 2.1% patients received chemotherapy and 1.7% patients received trastuzumab
Kim2018^27^	1.80%	48 (median)	3 patients experienced ipsilateral recurrences (2 patients LN− and 1 patient LNx)	NR
Magnoni2019^29^	1.90%	132 (median)	29 patients occurred recurrences (LN−, 17 local, 6 regional, 6 distant), 7 patients occurred recurrences (LN+, 4 local, 1 regional, 2 distant). 10‐year DFS 77.5%, 10‐year OS 94.8%	51.8% of negative SLN received systemic therapy and 74.2% of positive SLN received systemic adjuvant therapy
Bertozzi2019^30^	3.60%	NR	5‐year OS 85.71% (LN−), 5‐year OS 99.83% (LN+)	NR
Phantana2019^31^	1.10%	27 (median)	5‐years OS 100%	NR
Strang 2020^42^	NR	23 (median)	4 patients experienced recurrences (3 local and 1 distant)	28% patients received endocrine therapy and 5% received chemotherapy
Si 2020^43^	NR	61 (median)	9 patients experienced recurrences (4 local + region, 4 distant, 1 concurrent local and distant). 5‐year OS 99.36%	NR
Hacking2022^36^	2.30%	55 (median)	5‐year DFS 91.7%	NR
Kwon 2010^44^	NR	60.8 (median)	4 patients experienced recurrences (3 local and 1 distant). 5‐year RFS 97.4%	All recurrent patients did not receive systematic treatment

*Note*: Systemic adjuvant therapy included chemotherapy or (and) endocrine or (and) targeted therapy; Marco, lymph node macrometastasis.

Abbreviations: DFS, disease‐free survival; LN−, lymph node‐negative group; LN+, lymph node‐positive group; LNx, unknown lymph node status; NR, not reported; OS, overall survival; RFS, recurrence‐free survival.

In summary, axillary lymph node status was a crucial prognostic factor for breast cancer. The prognosis of breast cancer with ALNM was worse than that without metastasis. The results of our study suggested that the ALNM rate for DCIS‐MI is 8%. Thus, the determination the axillary lymph node status of DCIS‐MI was crucial for subsequent treatment decisions and prognosis evaluation.

### 4.2 Is it necessary to perform axillary lymph node dissection if sentinel lymph node biopsy is positive for subjects with DCIS‐MI?

In this group, the rates of axillary lymph node macrometastasis, micrometastasis, and ITC were 2%, 3%, and 2%, respectively. However, the conclusions regarding to the necessity for the performance of axillary lymph node dissection (ALND) was controversial. One study demonstrated that the omitting ALND for sentinel lymph node micrometastasis rather than ITC increased the risk of death and recurrence.^51^ Conversely, the 10‐year follow‐up results of IBCSG 23‐0110 demonstrated that patients with only sentinel lymph node micrometastasis (including ITC) showed no significant difference in DFS compared with or without ALND, suggesting ALND could be avoided for sentinel lymph node micrometastasis patients.^52^ And in our series, the Non‐SLN metastasis rate for patients with DCIS‐MI was as high as 33%; however, the rate of SLN macrometastasis was only 2% for patients with DCIS‐MI, with a good prognosis (Table 5). So we hypothesized that women with ALND could be exempted if SLN was negative or had 1–2 metastatic lymph node, which were consistent with the findings in NSABP B32,^53^ ACOSOG Z0011,^54^ and SENOMAC studies.^55^ Therefore, ALND may not be necessary if sentinel lymph node biopsy is positive for subjects with DCIS‐MI.

### 4.3 Risk factors for ALNM in subjects with DCIS‐MI

Regarding the risk factors or ALNM in DCIS‐MI, some studies association with age, vascular invasion, hormone receptor status, HER‐2 positivity, histological grade III, and tumor size ≥8 mm. For example, one study reported that DCIS tumor size ≥3.2 cm, combined with comedo and ER negativity, were independent risk factors for patients with DCIS‐MI.^27^ Another study observed that DCIS‐MI exhibited higher HER‐2 overexpression and lower ER and PR expression compared to DCIS.^34^ And no significant correlations with the factors abovementioned were observed in some studies as shown in Table 6. Therefore, it is important to note that these findings are derived from retrospective and small sample studies, highlighting the need for further prospective studies with large samples to validate these risk factors.

**TABLE 6:** Published literature search results on the *p*‐value of risk factors for axillary lymph node metastasis in DCIS‐MI.

Study	Age	ER (−)	HER‐2 (3+)	Ki‐67	LVI (+)	Nuclear grade (III)	Multifocal (2+)	Size of DCIS	Comedonecrosis
Pimiento 2011^15^	0.059	0.189	NR	NR	‐	0.54	NR	0.68	0.17
Ko 2012^16^	0.32 (<50)	0.03	0.46	NR	0.001	0.542	0.165	0.583 (>3 cm)	NR
Matsen 2014^23^	0.17	0.48	0.1	NR	0.01	0.78	0.95	NR	NR
Costarelli 2018^28^	NR	0.36	0.71	0.33 (>15%)	0.01	0.63	NR	0.47 (>5 cm)	NR
Holm 2019^7^	0.0002 (≤49)	0.9	0.03	NR	NR	NR	NR	0.11 (>2 cm)	NR
Gooch 2019^41^	0.001 (median 54)	0.485	0.976	NR	0.001	0.024	NR	0.003 (≥8 mm)	NR
Fan 2020^33^	0.0003 (<40)	NR	0.031	NR	NR	0.0001	NR	NR	NR
Paik 2022^35^	0.002 (<50)	0.001 (ER+)	0.09	0.091 (>20%)	0.001	0.174	NR	NR	NR

*Note*: Age, values greater than those in parentheses as a reference; ER (−), positive estrogen receptor (ER) as a reference; HER‐2 (3+), negative human epidermal growth factor receptor 2 (HER‐2) as a reference; LVI (+), negative vascular invasion as a reference; Multifocal (2+), single focal as a reference; Nuclear grade (III), nuclear grade I and (or) II as a reference; Size of DCIS, valuesless than those in parentheses as a reference.

The underlying reasons for the inconsistencies in the rates of ALNM in patients with DCIS‐MI was listed as the following: (1) the diversity in diagnostic methods for lymph node metastasis, 5% false‐positive by (H&E) staining in comparison with immunohistochemistry (IHC),^56^ a detection rate of 1.5% to 1.8% by (H&E) staining.^6^, ^31^ When compared to the 12.5% to 21.5% using HE staining combined with IHC.^7^, ^36^ This study aligns with this trend, confirming that the DCIS‐MI ALNM rate diagnosed by H&E staining combined with IHC was 9%, while H&E staining was 6%; (2) the diversity in detection methods of SLNB: The gold standard for SLNB in breast cancer involves using technetium‐99m (Tc‐99m) sulfur colloid under image guidance or Tc‐99 m sulfur colloid combined with blue dye, with a detection rate of 99.8%.^57^ This study aligns with existing views, indicating that when using technetium‐99 m (Tc‐99 m) sulfur colloid for axillary lymph node metastasis detection, the ALNM rate is the same as when using Tc‐99 m sulfur colloid combined with blue dye (12% vs. 11%); (3) the differences types of research included: Compared with prospective studies, the conclusions drawn from retrospective studies lack reliable scientific validity.^58^ In this study, the axillary lymph node metastasis rate was 7% in the retrospective research, while it was 12% in the prospective research. However, whether the axillary lymph node metastasis rate for DCIS‐MI is consistently higher in prospective studies than in retrospective studies requires confirmation from large‐scale clinical trials; (4) the discrepancy in the baseline characteristics of enrolled patients: such as the differences in the histological grade,^33^ age at diagnosis HER‐2 status,^7^ hormone receptor negativity, multi‐lesions,^41^ race and ethnicity, tumor size ≥8 mm, and lymphatic vessel invasion (LVI).

In summary, the inconsistent ALNM rates in the included DCIS‐MI studies may be attributed to the above reasons, possibly contributing to the strong heterogeneity in this article. However, this study performed subgroup analyses based on study types, SLNB development staining techniques, lymph node histological examination, data sources, study quality, region‐wise, tumor grading classification, classification of DCIS combined with necrosis, classification of DCIS types, classification of vascular infiltration and time span. No significant heterogeneity factors were identified, which may be related to the excessive absence of basic characteristics in the included literature patients (50% of the SLNB development staining techniques were unknown, 25% of lymph nodes histological examinations were unknown, 21.4% of tumor grading classifications were unknown, 71.4% of DCIS combined with necrosis classifications were unknown, 71.4% of DCIS types classifications were unknown, and 64.3% of vascular infiltration classifications included were unknown).

### 4.4 Limitations

1. Significant heterogeneity. Many enrolled patients are from retrospective studies, resulting in nonuniform clinical characteristics and numerous missing patient details, impeding significant reduction through subgroup analysis. Variables like higher tumor histological grading, younger age, estrogen negativity, HER‐2 positivity, and LVI could contribute to increased ALNM rates in DCIS‐MI patients. The intention to conduct subgroup analysis based on the proportion of these factors is hindered by the lack of a defined proportion supported by evidence‐based medicine.
2. Large inclusion time span (1989–2020): This period introduces variability in breast cancer treatment methods (surgical methods and concepts, chemotherapy, targeted therapy, endocrine therapy, etc.) and diagnostic practices, limiting the ability to account for evolving therapies and changes in DCIS‐MI diagnosis and prognosis.

## 5 CONCLUSION

The SLNB for patients with DCIS‐MI is justifiable and could provide a novel therapeutic basis for systemic treatment decisions. Future researched should focus on identifying subgroups with higher Non‐SLN involvement risk and determining which DCIS‐MI patients might benefit from additional adjuvant therapy.

## AUTHOR CONTRIBUTIONS

**Xiongxiong Li:** Conceptualization (lead); data curation (lead); formal analysis (lead); investigation (lead); methodology (lead); project administration (lead); resources (lead); software (lead); supervision (lead); validation (lead); visualization (lead); writing – original draft (lead); writing – review and editing (lead). **Can Zhou:** Conceptualization (equal); data curation (lead); formal analysis (lead); investigation (equal); methodology (lead); project administration (equal); resources (equal); software (lead); supervision (equal); validation (equal); visualization (equal); writing – original draft (lead); writing – review and editing (lead). **Ting Xu:** Conceptualization (equal); data curation (lead); formal analysis (lead); investigation (equal); methodology (equal); project administration (equal); resources (equal); software (lead); supervision (equal); validation (equal); visualization (equal); writing – original draft (supporting); writing – review and editing (supporting). **Yu Ren:** Conceptualization (equal); data curation (supporting); formal analysis (supporting); investigation (equal); methodology (supporting); project administration (equal); resources (equal); software (lead); supervision (equal); validation (equal); visualization (equal); writing – original draft (supporting); writing – review and editing (supporting). **Meng Li:** Conceptualization (supporting); data curation (supporting); formal analysis (supporting); investigation (supporting); methodology (supporting); project administration (supporting); resources (supporting); software (supporting); supervision (supporting); validation (supporting); visualization (supporting); writing – original draft (supporting); writing – review and editing (supporting). **Jin Shang:** Conceptualization (equal); data curation (equal); formal analysis (equal); funding acquisition (equal); investigation (equal); methodology (equal); project administration (equal); resources (equal); software (equal); supervision (equal); validation (equal); visualization (equal); writing – original draft (equal); writing – review and editing (equal).

## CONFLICT OF INTEREST STATEMENT

The authors declare no conflicts of interest.

## Supporting information


Appendix S1.

## Data Availability

The literatures and/or datasets used and/or analyzed during the current study are available from the corresponding author on reasonable request.
