# Supplementary figures and images for: Meta‐analysis on axillary lymph node metastasis rate in ductal carcinoma in situ with microinvasion
Source: Cancer Med. 2024 Jun 26;13(12):e7413. doi: 10.1002/cam4.7413 (PMC11199912; doi:10.1002/cam4.7413)

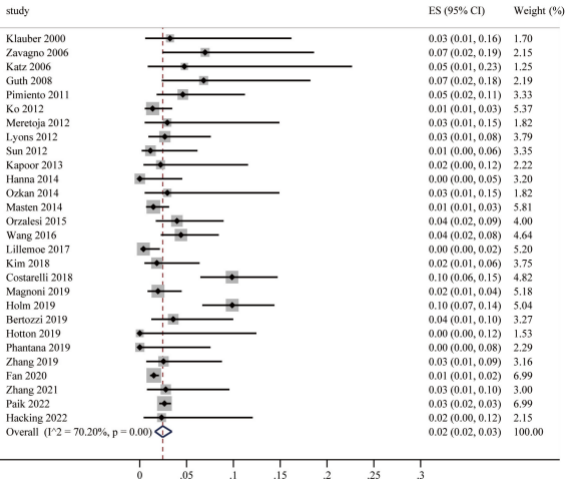

Supplement: Supplementary file 1 — Appendix S1. [file CAM4-13-e7413-s001.zip › Figure S1a Forest plot of macrometastasis in DCIS-MI.pdf]

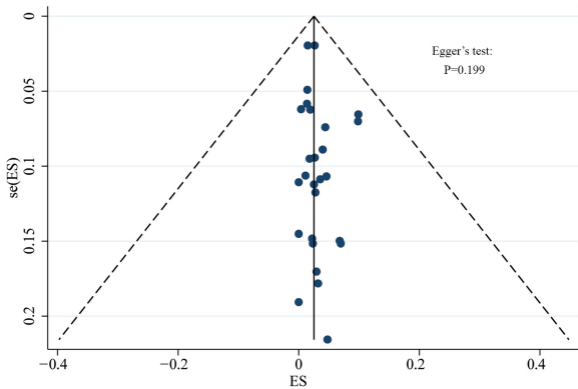

Supplement: Supplementary file 1 — Appendix S1. [file CAM4-13-e7413-s001.zip › Figure S1b Funnel plot of macrometastasis in DCIS-MI.pdf]

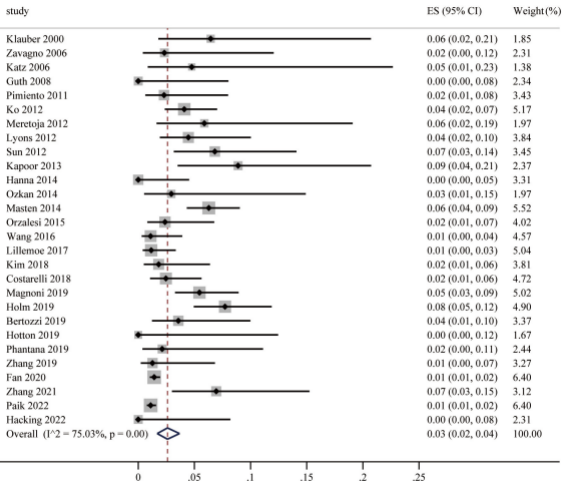

Supplement: Supplementary file 1 — Appendix S1. [file CAM4-13-e7413-s001.zip › Figure S2a Forest plot of micrometastasis in DCIS-MI.pdf]

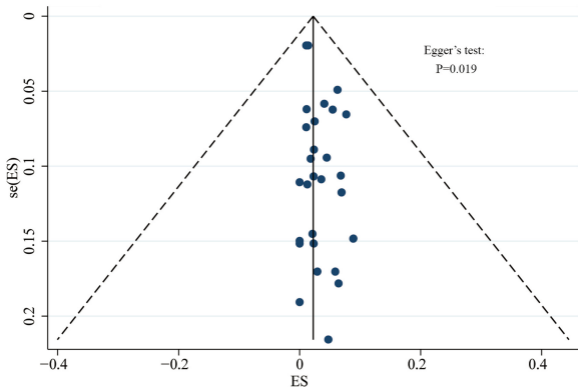

Supplement: Supplementary file 1 — Appendix S1. [file CAM4-13-e7413-s001.zip › Figure S2b Funnel plot of micrometastasis in DCIS-MI.pdf]

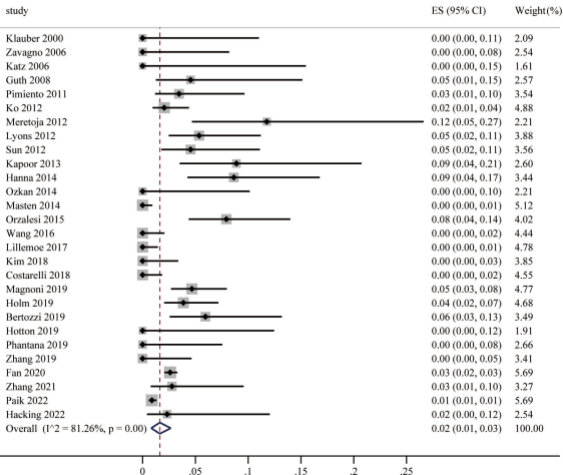

Supplement: Supplementary file 1 — Appendix S1. [file CAM4-13-e7413-s001.zip › Figure S3a Forest plot of ITC in DCIS-MI.pdf]

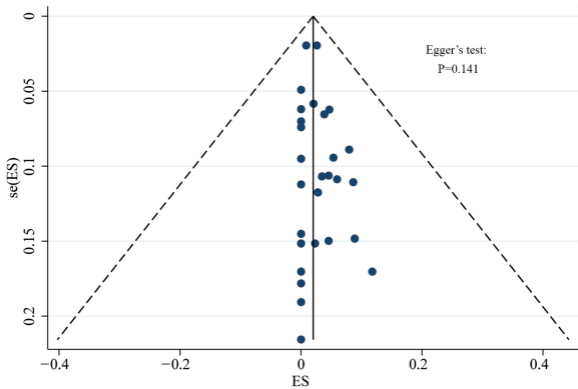

Supplement: Supplementary file 1 — Appendix S1. [file CAM4-13-e7413-s001.zip › Figure S3b Funnel plot of ITC in DCIS-MI.pdf]
